# Potential Interplay of the Gatipotuzumab Epitope TA-MUC1 and Estrogen Receptors in Ovarian Cancer

**DOI:** 10.3390/ijms20020295

**Published:** 2019-01-12

**Authors:** Sabine Heublein, Sabina Page, Doris Mayr, Elisa Schmoeckel, Fabian Trillsch, Frederik Marmé, Sven Mahner, Udo Jeschke, Aurelia Vattai

**Affiliations:** 1 Department of Obstetrics and Gynecology, Ludwig-Maximilians University of Munich, University Hospital, 81377 Munich, Germany; sabinakp@aol.com (S.P.); fabian.trillsch@med.uni-muenchen.de (F.T.); sven.mahner@med.uni-muenchen.de (S.M.); udo.jeschke@med.uni-muenchen.de (U.J.); aurelia.vattai@med.uni-muenchen.de (A.V.); 2 Department of Obstetrics and Gynecology, University of Heidelberg, 69117 Heidelberg, Germany; frederik.marme@med.uni-heidelberg.de; 3 Department of Pathology, Ludwig-Maximilians University of Munich, 81377 Munich, Germany; doris.mayr@med.uni-muenchen.de (D.M.); elisa.schmoeckel@med.uni-muenchen.de (E.S.)

**Keywords:** Gatipotuzumab, Tamoxifen, ovarian cancer, 4-Hydroxy-Tamoxifen (4-OHT), survival

## Abstract

Anti-tumor efficacy of Gatipotuzumab, a therapeutic antibody targeting Tumor-Associated Mucin-1 (TA-MUC1), in relapsed ovarian cancer (OC) appeared to be rather heterogeneous. Whether adding a second anti-neoplastic drug may augment response towards Gatipotuzumab, has not been elucidated so far. Since it is known that anti-MUC1 antibodies may alter estrogen receptor activity in breast cancer, this potential interplay was investigated in OC. The correlation between TA-MUC1, estrogen receptors (ERs) and another 12 protein markers as well as their correlation with clinico-pathological parameters in 138 ovarian cancer cases was studied. Finally, Gatipotuzumab and 4-Hydroxy-TTamoxifen (4-OHT) as well as the combination of both was tested for its impact on cell viability in COV318, OV-90, OVCAR-3, and SKOV-3 cells. A strong positive correlation between TA-MUC1 and ERs was detected in OC tissue. Those cases missing ERs but staining positive for TA-MUC1 had significantly reduced overall survival. The combination of 4-OHT and Gatipotuzumab significantly reduced cell viability and was more effective than treatment with Gatipotuzumab alone. Co-stimulation with Gatipotuzumab enhanced the efficacy of 4-OHT in OVCAR-3 and SKOV-3. The data suggest an interplay of TA-MUC1 and ERs in OC. Whether the combination of Gatipotuzumab and TTamoxifen may enhance efficacy of either of the two drugs *in vivo*, or may even translate into a clinically relevant benefit over the respective monotherapies, remains to be investigated.

## 1. Introduction

MUC1 is an abundantly expressed transmembrane glycoprotein that undergoes several glyco-modifications during the process of malignant transformation, leading to the exposure of tumor-specific, novel carbohydrate epitopes [1]. Targeting these neo-epitopes seems to be attractive in terms of generating effective, highly selective, and well tolerated anti-cancer therapeutics. Gatipotuzumab has been designed to recognize such a cancer specific, conformational carbohydrate epitope on MUC1 with exceptional high specificity and affinity [2,3]. Structure-wise, this epitope is built of the amino acid motif PDT*RP motif where T* stands for a specifically *O*-glycosylated threonine residue [3,4]. Due to its tumor selectivity, the epitope of Gatipotuzumab has been termed tumor-associated MUC1 (TA-MUC1). TA-MUC1 is present in a wide variety of cancer entities but virtually absent in normal, non-neoplastic tissue [5,6]. This also applies for ovarian cancer (OC) as shown by an earlier study of our group [7].

So far, two clinical trials have tested Gatipotuzumab for its efficacy in patients with advanced cancer. Fiedler et al. recently published a phase I dose-escalation trial where Gatipotuzumab was applied to patients with advanced cancer, who had failed to respond to standard therapy any longer [8]. Patients, among them 20 women diagnosed for OC, had progressive disease at study entry. The authors report a clinical benefit in 28 out of 60 patients treated with Gatipotuzumab. Tumor control activity of the antibody seemed to be particularly high in a patient with highly pretreated OC [8]. A second study evaluated Gatipotuzumab as a maintenance therapy in OC [9]. This randomized, placebo controlled phase 2 study—though not fully published yet—reported that Gatipotuzumab did not alter outcome of patients when applied as a maintenance therapy for relapsed OC [9]. 

In general, two main Gatipotuzumab mediated anti-tumor modes of action have been investigated. First, the murine precursor of Gatipotuzumab as bound to TA-MUC1 induces antibody-dependent-cell-mediated cytotoxicity (ADCC) and phagocytosis (ADCP) leading to cytolysis of the cancer cell [2]. Meanwhile immunogenicity of the antibody has been significantly enhanced by so called glyco-optimization using glyco-engineered production cell lines on the GlycoExpress^TM^ platform. A “human-like” glycosylation of the now fully humanized antibody increases immunogenicity of the antibody plus TA-MUC1 complex and hence, triggers a stronger ADCC/ADCP response ([8] and data on file; Glycotope GmbH, Berlin, Germany). Second, Gatipotuzumab becomes internalized into the cancer cell by endocytosis once bound to TA-MUC1 [2]. Thus, drug conjugates of the antibody can be utilized to shuttle toxins or radionuclides into cancer cells.

As a third mechanism of action, anti-MUC1 antibodies directly influence MUC1 intracellular downstream signaling pathways, thereby altering sensitivity towards cytostatic drugs in OC cells and xenografted mice [10,11]. In line with this, induction of endogenous anti-MUC1 antibodies by vaccination enhanced response towards endocrine therapy in patients with metastatic breast cancer [12,13,14]. Additionally, MUC1 itself has been implemented to induce Tamoxifen resistance, again supporting the existence of a crosslink of MUC1 and estrogen signaling [15,16]. Whether targeting MUC1 by Gatipotuzumab may reconstitute or even enhance sensitivity towards Tamoxifen has not been studied so far. Accordingly, it is unknown whether addition of Tamoxifen may alter anti-tumor efficacy of Gatipotuzumab. Therefore, the current study was designed to investigate whether a statistical or functional crosslink of TA-MUC1 and estrogen receptors might exist in OC. Furthermore, we aimed to investigate whether targeting TA-MUC1 by Gatipotuzumab may alter efficacy of Tamoxifen in OC cells and vice versa.

## 2. Results

### 2.1. TA-MUC1 as Stained by Gatipotuzumab Correlates with Estrogen Receptor Expression

The majority of cases analyzed (Table 1) had been diagnosed with ovarian cancer of advanced FIGO (Fédération Internationale de Gynécologie et d’Obstétrique) stage (FIGO III/IV in 93/133 patients (69.9%)). Information on lymph node resection was available from 84 patients and cancer spread to lymph nodeswas found in 56.0% (47/84) of these cases. High grade cancer was diagnosed in 95/126 (75.4%) of patients. The majority of tumors were found to be of serous histology (68.8%, 95/138).

Presence of the Gatipotuzumab antigen TA-MUC1 was positively correlated to expression of both ERα and GPER (Table 2). No correlation was detected in terms of TA-MUC1 and ERβ. Furthermore, TA-MUC1 was positively associated with Glycodelin as stained by two anti-Glycodelin antibodies that detect the protein backbone of Glycodelin. No correlation was observed in the case of the immunosuppressive glycovariant Glycodelin A. Further, TA-MUC1 was not correlated to HER2. As expected, TA-MUC1 was correlated to MUC1 as stained with different antibodies (115D8, HMFG1, VU3C6, VU4H8) designed to detect protein epitopes on MUC1.

### 2.2. Combined Expression Patterns of TA-MUC1 and Estrogen Receptors

**Figure 1:**
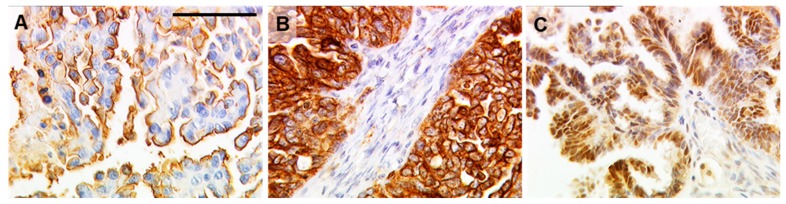
Immunostaining of TA-MUC1, GPER, and ERα in ovarian cancer tissue. Representative photomicrographs of TA-MUC1 (as detected by Gatipotuzumab; (**A**)), GPER (**B**) and ERα (**C**) are shown. The scale bar in A equals 100 µm and refers to (**A**–**C**).

The study sample was split up by a four-dimensional staining score recognizing double positive, double negative, solely TA-MUC1 positive and solely GPER or ERα positive cases, respectively. Representative images of TA-MUC1, GPER, and ERα staining in OC tissue are presented in Figure 1.

Co-expression of TA-MUC1 and GPER was detected in 94 (68.1%) cases, while 23 (16.7%) and 15 (10.9%) samples expressed either solely TA-MUC1 or solely GPER, respectively. Six (4.3%) patients were found to be double negative. Co-expression of TA-MUC1 and GPER (TA-MUC1^pos^ + GPER^pos.^) was significantly more common in serous OC (*p* = 0.004) but was not related to the remaining pathological parameters. Presence of TA-MUC1 and at the same time loss of GPER (TA-MUC1^pos^ + GPER^neg.^) characterized a subgroup of patients that were significantly more often staged as FIGO III/IV (*p* = 0.004) as compared to the remaining cases. In addition, patients with this staining pattern were more often diagnosed with positive lymph nodes (*p* = 0.024) or high-grade cancer (*p* = 0.004) (Table 3).

Regarding ERα, a number of 6 (4.3%) cases was found to solely express ERα, while 79 (57.2%) only expressed TA-MUC1. Double positivity for ERα and TA-MUC1 was observed in 38 (27.5%) cases. Just 15 (10.9%) samples expressed neither ERα nor TA-MUC1. Clinicopathological characteristics as split up by the four-dimensional TA-MUC1—ERα scores were tested for statistical associations. Co-expression of TA-MUC1 and ERα was not statistically associated to the clinicopathological data tested. TA-MUC1 positivity without co-expression of ERα was more common in tumors of serous histology (*p* = 0.037) or high grade (*p* = 0.007) (Table 3).

The IHC staining pattern TA-MUC1^pos^ + GPER^neg^ + ERα^neg^, i.e., expression of TA-MUC1 without co-expression of any of the two ERs, was detected in 18 cases and was associated with advanced FIGO stage (*p* = 0.004), cancer spread to retroperitoneal lymph nodes (*p* = 0.021) and poor histologic differentiation (*p* = 0.011) (Table 3).

**Figure 2:**
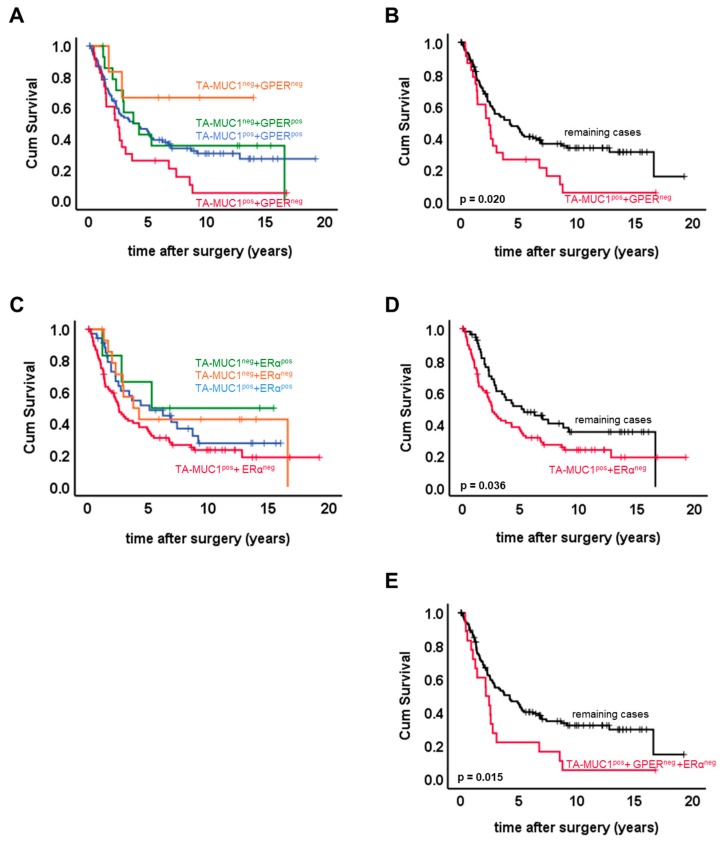
Overall survival (OS) of ovarian cancer patients split up by TA-MUC1/GPER and TA-MUC1/ERα staining patterns. Patients’ OS was split up according to their TA-MUC1/GPER staining patterns (**A**). Cases expressing TA-MUC1 but at the same time staining negative for GPER were found to have significantly shortened OS (**B**). The same applied for patients positive for TA-MUC1 but not expressing ERα (**D**), though pairwise comparison of OS among TA-MUC1/ERα staining patterns did not reveal significant differences (**C**). Finally, patients solely expressing TA-MUC1 but neither of the two ERs (GPER^neg^ and ERα^neg^) had significantly shortened OS as compared to remaining cases (**E**).

Staining patterns were contrasted regarding patients’ OS (overall survival) (Figure 2A). Within pairwise comparisons, those patients only staining positive for the Gatipotuzumab epitope were identified with significantly reduced OS as compared to both double negative cases (*p* = 0.022) and to the whole group of remaining cases (*p* = 0.02) (Figure 2A, B). Four-dimensional patterning of TA-MUC1/ERα expression was performed accordingly (Figure 2C). Patients solely expressing TA-MUC1 without co-expressing ERα had significantly decreased OS as compared to the whole group of remaining cases (*p* = 0.036; Figure 2D). Finally, absence of both ERs (ERα and GPER) in TA-MUC1 positive patients turned out to be a staining pattern associated with markedly reduced OS (*p* = 0.015; Figure 2E). None of the four staining patterns introduced above was of prognostic significance within multivariate analysis.

### 2.3. Combination of Tamoxifen and Gatipotuzumab Reduces Viability of OC Cell Lines

**Figure 3:**
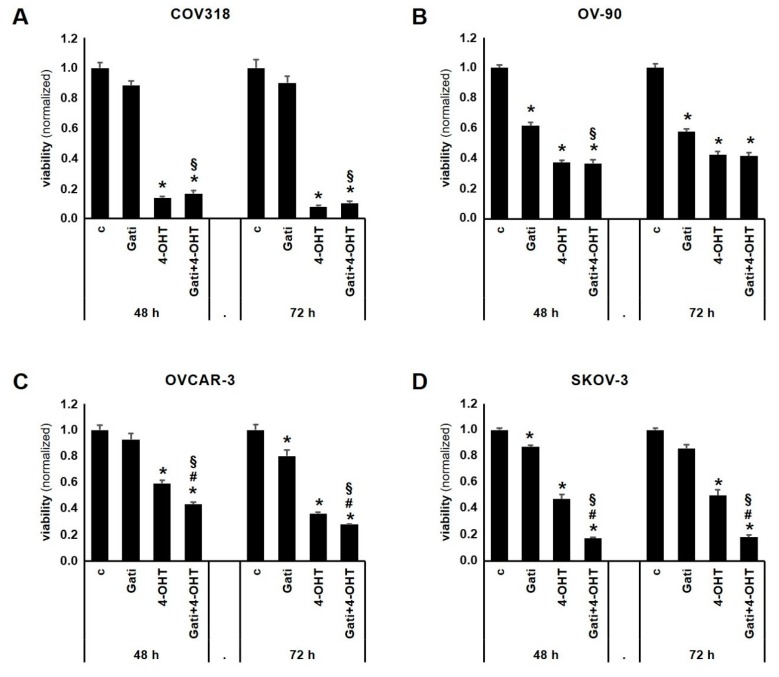
Impact of Gatipotuzumab and 4-Hydroxy-Tamoxifen (4-OHT) on OC viability. Stimulation with Gatipotuzumab, 4-OHT and the combination of the two was performed in COV318, OV-90, SKOV-3, and OVCAR-3 cell lines over a 48-h and 72-h period. In general, Gatipotuzumab (Gati) alone only moderately reduced cell viability, while 4-OHT treatment appeared to be efficient in all the four cell lines tested (**A**–**D**). When viability of cells that had undergone co-stimulation was compared to that of those that had been treated with a single substance only, the overall net effect of co-stimulation was numerically slightly higher than the sum of individual effects of any of the two substances alone OVCAR-3 (**C**) and SKOV-3 (**D**). However, this was not statistically significant. Significant changes are indicated as follows: “*” equals significant reduction as compared to “control”; “§” indicates significant reduction as compared to samples treated with Gatipotuzumab alone; “#” indicates significant reduction as compared to cells treated with 4-OHT alone.

**Figure 4:**
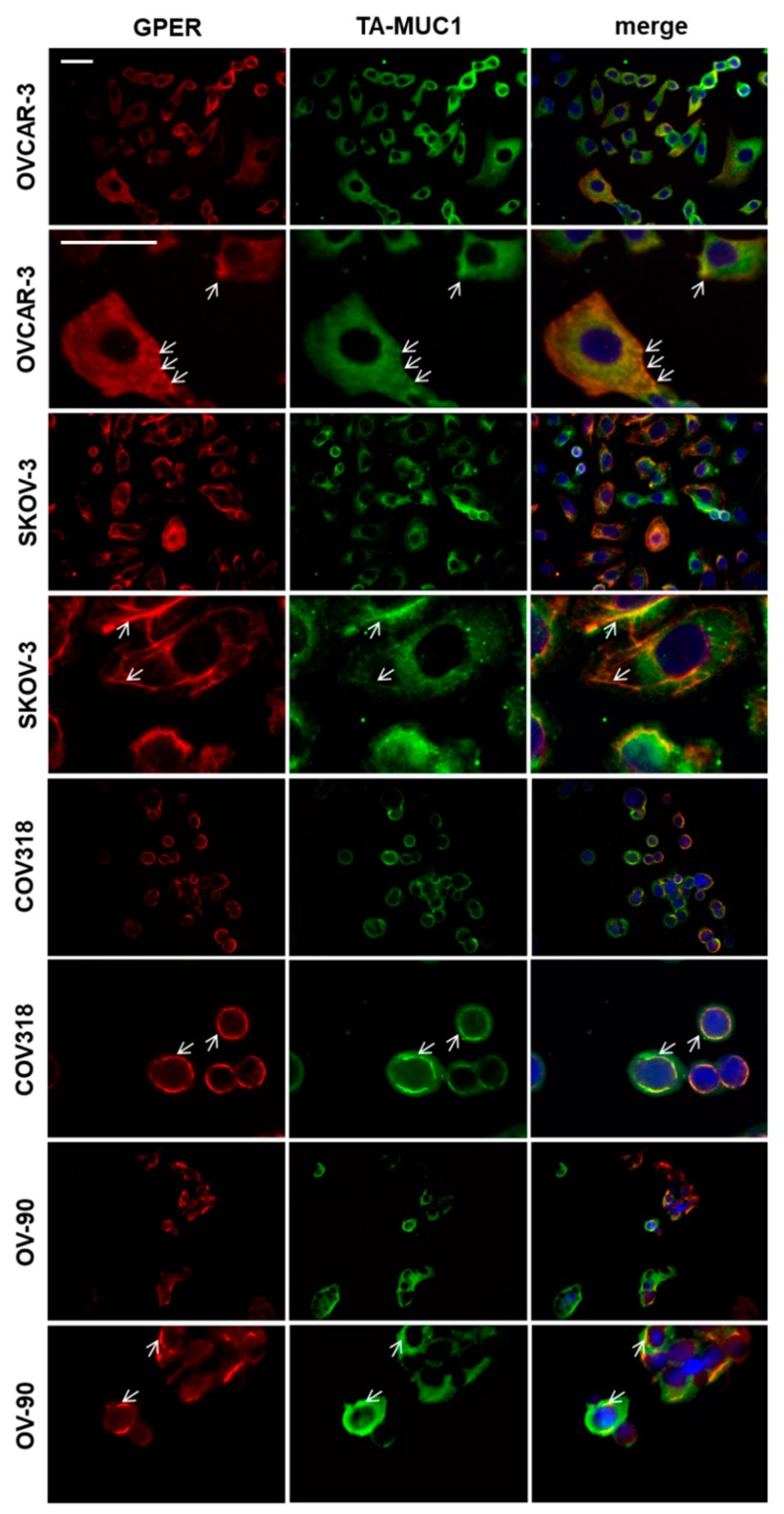
Co-staining of TA-MUC1 and GPER in ovarian cancer cell lines. Double immunofluorescence staining of GPER and TA-MUC1 in SKOV-3, OVCAR-3, COV318, and OV-90 ovarian cancer cell lines identified co-expression of the two proteins. Arrows indicate focal co-localization of TA-MUC1 and GPER. Scale bars represent 50 µm and apply to each upper and each lower panel, respectively. Representative images are presented.

OVCAR-3, SKOV-3, OV-90, and COV318 cells were employed to evaluate the effect of Gatipotuzumab, 4-Hydroxy-Tamoxifen (4-OHT), and the combination of the two on cell viability (Figure 3). TA-MUC1, ERα, and GPER were expressed in all the four cell lines (Figure 4 and [17,18,19]). Gatipotuzumab alone clearly reduced viability in OV-90 (48 h: 0.62-fold, *p* = 0.006; 72 h: 0.58-fold, *p* = 0.006) but was less efficient in the remaining cell lines. 4-OHT, which is the active metabolite of Tamoxifen, has been demonstrated to act as a selective estrogen receptor modulator on ERα and as an activator of GPER. Viability of all cell lines was significantly reduced by 4-OHT at both timepoints. 4-OHT as a single agent proved to be most potent in COV318 at the 72 h timepoint yielding a reduction of cell viability down to 0.08 of control level (*p* < 0.001). Finally, co-stimulation using both Gatipotuzumab and 4-OHT was performed over a 48 h and 72 h period, respectively. Again, co-stimulation revealed a significant reduction of viability in all the four cell lines tested with the strongest effect (as compared to control) being present in COV318 at 72 h (0.1-fold, *p* < 0.001). Viability of cells that had undergone co-stimulation was compared to those that had been treated with a single substance only. The combination of 4-OHT and Gatipotuzumab significantly reduced viability of all cell lines as compared to samples that had been treated with Gatipotuzumab alone (Figure 3A–D). Except for OV-90 at the 72 h timepoint, this effect was statistically significant at both time points tested. Regarding SKOV-3 and OVCAR-3 the combination of 4-OHT and Gatipotuzumab was superior compared to 4-OHT alone (48 h: SKOV-3: *p* = 0.021; OVCAR-3: *p* = 0.001; 72 h: SKOV-3: *p* = 0.014; OVCAR-3: *p* = 0.048). In OVCAR-3 and SKOV-3 the overall net effect of 4-OHT and Gatipotuzumab co-stimulation was numerically slightly higher than the sum of individual effects of any of the two substances alone (OVCAR-3: 48 h: 9.9%; SKOV-3:48 h: 19.5%; 72 h: 17.9%). However, this difference was not significant.

Cell lines were co-stained for TA-MUC1 and GPER. Both proteins were found to be co-expressed in the large majority of cells. TA-MUC1 showed a uniform cytoplasmic staining while GPER was found to be distributed in a web like cytoplasmic pattern. Both TA-MUC1 and GPER showed partial membrane staining, especially in OVCAR-3 and SKOV-3 (Figure 4). Regarding these two cell lines, subcellular co-localization of the two was observed mostly in focal condensations at the cell membrane (Figure 4).

## 3. Discussion

This study found the Gatipotuzumab epitope TA-MUC1 to be closely correlated to ER expression in OC tissue. Furthermore, when OC cell lines were exposed to Gatipotuzumab and Tamoxifen, the combination of both drugs significantly decreased cell viability in all the four cell lines tested and was superior as compared to Gatipotuzumab alone. Further, co-stimulation with Gatipotuzumab enhanced the efficacy of 4-OHT in OVCAR-3 and SKOV-3. 

Both GPER and ERα were closely correlated to membrane expression of TA-MUC1 in the patient cohort investigated in the current study. Although a statistical correlation by far does not prove functional interaction, it may suggest a potential crosstalk of TA-MUC1 and ERs. As GPER and TA-MUC1 are both localized on the cell membrane and in the cytoplasm, we performed double immunofluorescence and found co-localization of the two. Though a potential physical interaction would require this finding to be backed up by e.g., immunoprecipitation or a proximity ligation assay, we herein provide initial evidence of a potential interplay of TA-MUC1 and GPER. Since both drugs have already been administered to OC patients in clinical trials, a potential functional interaction of the two might be of clinical interest. Hence, further research should be done thus to clear whether a functional or even physical interaction of TA-MUC1 and GPER may exist. There are several lines of evidence supporting this hypothesis. With respect to signaling pathways, the C-term of MUC-1 was found to detach from the cell membrane and to translocate to the nucleus. Nuclear MUC-1-C binds ERα, thus attenuating Tamoxifen induced changes on ERα dependent transcription [20]. As a second mechanism, cell surface MUC-1 directly interacts with receptor tyrosine kinases and may also interfere with their downstream pathways [21,22,23]. In line with this, anti-MUC1 antibodies have already been shown to inhibit EGF receptor signaling in cancer cells [10]. We therefore tested TA-MUC1 for correlation to HER2 and found no significant association of the two. However, we did not stain for other members of the EGFR (epidermal growth factor receptor) family. Thirdly, GPER is known to even physically interact with EGFR as demonstrated by immunoprecipitation experiments [24]. Furthermore, GPER signaling—at least in part—seems to be transduced via the EGFR-MAPK-ERK pathway as well. However, the interaction of GPER with the MAPK signaling axis seems to be context or cell type dependent. While some authors report that GPER activates EGFR signaling and thereby acts as an oncogene, others found that constitutive activation of this pathway as mediated by GPER inhibits cell proliferation [25,26]. Since especially OVCAR-3 and SKOV-3 have been reported to express high levels of EGFR and to be responsive to the blockade of EGFR regulators, their sensitivity towards the combined blockade of TA-MUC1 and GPER may—at least partly—be related to their dependency on EGFR [27].

Tamoxifen is known to activate GPER and to selectively modulate ERα [28]. The current study revealed an anti-proliferative effect of Tamoxifen treatment on ovarian cancer cell lines. A similar activity of Tamoxifen on ER positive OC cells had been described before and could be reproduced in OC cells stimulated with the GPER selective agonist G1 [29]. The exact mechanism underlying this phenomenon has not been described so far. Rather than elucidating the molecular details that cause antiproliferative actions of Tamoxifen, the current study questioned whether targeting ERs with Tamoxifen may influence efficacy of Gatipotuzumab in OC cells. Like Tamoxifen, MUC1 interacting antibodies are known to inhibit growth in various cancer models including OC [10,11]. So far, the effect of Gatipotuzumab on OC cell viability has not been published. The in vitro data presented above demonstrate that Gatipotuzumab reduces OC viability to a rather moderate extent. Importantly, these data do not adequately reflect the efficacy of Gatipotuzumab in an *in vivo* model of the disease or in humans since, e.g., immune related anti-tumor effects—an important pillar of Gatipotuzumab’s mode of action—are fully excluded in a 2D cell culture setup. Anyhow, as the aim of the current study was to elucidate potential interplay of TA-MUC1 and ERs, the cell culture system was judged to be the most straight forward and most adequate model to pursue this question.

Interestingly, the anti-proliferative activity of Gatipotuzumab could be enhanced by co-stimulation with Tamoxifen. Regarding the OVCAR-3 and SKOV-3 cell lines, the anti-proliferative action of both drugs in combination was greater than the sum of individual effects of any of the two drugs alone—though this was not statistically significant. Since such effects were only observed in two out of four cell lines, they need to be interpreted with care. Whether Tamoxifen indeed facilitates action of Gatipotuzumab (and vice versa) remains to be proven by independent experiments and *in vivo* models. Furthermore, TA-MUC1, GPER, or ERα negative OC cell lines, knock down models and analysis of downstream pathways need to be employed thus to investigate potential interplay of the three proteins from a mechanistic point of view. Nevertheless, correlation and co-localization of ERs and TA-MUC1 suggests that the effects observed in SKOV-3 and OVCAR-3 may be caused by direct or indirect crosstalk of TA-MUC1 and ERs.

The current study detected patients solely expressing TA-MUC1 to have particularly shortened OS as compared to remaining subgroups. This association was lost within multivariate analysis. In line with this, presence of TA-MUC1 without co-expression of ERs (TA-MUC1^pos^ + ERα^neg^ + GPER^neg^) was positively correlated to advanced FIGO stage, high grade and cancer spread to retroperitoneal lymph nodes (Table 3). A former study from our group reports that TA-MUC1 as detected by Gatipotuzumab was not related to patient outcome in the case the patient cohort had not been stratified for ERs [7]. We herein demonstrated that TA-MUC1 and VU4H5 IHC scores were closely associated. VU4H5 is counted among the most widely used anti-MUC1 antibodies and presence of total MUC1, as detected by the monoclonal mouse anti-MUC1 antibody VU4H5, was associated with shortened overall survival in the same, non-stratified patient panel [30]. Interestingly, absence of either GPER or ERα and at the same time expression of TA-MUC1 made TA-MUC1 to function as a negative prognosticator for patients’ OS (Figure 2), too. Whether this observation might indeed be caused by an interaction of ERs with specifically glyco-modified MUC1, i.e., TA-MUC1, remains to be determined. So far, no data on TA-MUC1 interaction in OC have been published. Our finding described above supports the hypothesis that co-expression of ERs may alter tumor-biologic effects of TA-MUC1. Whether the rather heterogeneous response towards Gatipotuzumab monotherapy observed in clinical trials may be due to TA-MUC1 interacting proteins remains to be elucidated. However, our data suggest that ERs may alter TA-MUC1 dependency of cancer cells.

## 4. Materials and Methods

### 4.1. Study Panel

This study investigated the statistical and functional interaction of TA-MUC1 and ERs. Immunostaining of Gatipotuzumab, ERα, ERβ, and GPER as single, separate markers has been published before [7,29,31]. Data of another 12 protein biomarkers were retrieved from archival data published before and were correlated to Gatipotuzumab [7,29,30,31,32,33,34].

A total of 138 ovarian cancer tissue samples were available for studying the interplay of TA-MUC1 and GPER/ERα as well a potential prognostic value of marker combinations containing TA-MUC1. Formalin-fixed, paraffin-embedded (FFPE) tissue was retrieved from the histopatholgical archive of the Department of Gynaecology and Obstetrics, Ludwig-Maximilians-University, Munich, Germany. Tissue had been collected from 138 patients who had undergone surgery for OC from 1993–2002 at Department of Gynaecology and Obstetrics, Ludwig-Maximilians-University, Munich, Germany. Clinicopathological data were gathered from patients’ charts, pathology reports or from the Munich Cancer Registry and are displayed in Table 1 and explained in the results section. Median overall survival (OS) of the cohort was 3.30 years (95% CI: 2.03–4.57) and median follow up was 11.12 years (95% CI: 8.63–13.6). Study end point was OS and 92 deaths were observed during the follow up period. This study has been performed and presented according to the REMARK (Reporting recommendations for tumor marker prognostic studies) criteria for reporting biomarker prognostic studies [35].

### 4.2. Ethical Approval

The tissue samples used for the current analysis were retrieved from the archive of the Department of Gynaecology and Obstetrics, Ludwig-Maximilians-University, Munich, Germany. Tissue had been collected for routine diagnostics at the time the patients had been treated at our institution (1990–2002). When this retrospective study was initiated all diagnostic procedures had already been fully completed, the tissue samples were classified as left-over material and underwent irreversible anonymization. Under these circumstances no individual written informed consent was needed as per declaration of the Ethics Committee of the Ludwig-Maximilians-University. The study was approved by the Ethics Committee of the Ludwig-Maximilians-University (approval number 227-09 (amendment approved on 2 September 2011) and 18-392 (approved on 25 June 2018)) and was performed according to the standards set in the declaration of Helsinki 1975. All researchers were blinded from patient data during experimental analysis.

### 4.3. Immunohistochemistry

Immunohistochemical data of TA-MUC1 and GPER/ERα staining regarding this panel were retrieved from the laboratory archive as previously published [7,31,36]. An established semi-quantivative immunoreactive score (IRS) [7,37,38] was applied in order to quantify immunoreactivity. Staining scores were binarized thus to distinguish samples classified as TA-MUC1 negative vs. positive and GPER/ERα negative vs. positive, respectively. TA-MUC1 immunoreactivity at the cell membrane and in the cytoplasm were scored separately. Since membrane staining was much more prominent, membrane staining of TA-MUC1 was chosen over cytoplasm staining when interaction with GPER and ERα was tested for prognostic significance. A four-dimensional score was applied to distinguish cases staining positive for GPER only (TA-MUC1^neg^ + GPER^pos^), for TA-MUC1 only (TA-MUC1^pos^ + GPER^neg^), for both biomarkers (TA-MUC1^pos^ + GPER^pos^) or for neither of the two (TA-MUC1^neg^ + GPER^neg^). The same was done in case of ERα, respectively. Finally, the feature TA-MUC1^pos^ + GPER^neg^ + ERα^neg^ was studied.

### 4.4. Cell Culture

COV318, OV-90, OVCAR-3, and SKOV-3 were purchased form the American Tissue Culture Collection (ATCC) and from the European Collection of Cell Cultures (ECACC). All cell lines were grown in DMEM containing stable l-glutamine. Cell culture media was supplemented with 10% *v/v* fetal calf serum (Biochrom, Munich, Germany) and contained no antibiotics and antimycotics. Cell lines were routinely checked for absence of mycoplasma contamination by performing a commercially available PCR based mycoplasma test kit (AppliChem, Darmstadt, Germany). Cells were cultured in a humidified atmosphere at 37 °C and 5% CO_2_.

### 4.5. Double Immuno-Fluorescence

Cells were seeded on glass slides in quadriperm dishes. After a 36 h incubation period, attached cells were fixed in acetone solution for 5 min. Acetone was discarded and slides were washed three times in PBS. Cells were then blocked with Ultra V-Block for 15 min. Primary antibodies were applied at the following concentrations: rabbit anti GPER (Lifespan Biosiences, Seattle, WA, USA) 1:400, Gatipotuzumab (fully humanized, Glycotope, Berlin, Germany) 1:400. Following an incubation period of 16 h at 4 °C, slides were washed using PBS and incubated with secondary antibodies (goat anti rabbit IgG Cy3, goat anti human IgG + IgM biotinylated). Secondary antibodies were diluted 1:500 and 1:250, respectively and incubated for 30 min in the dark. Following washes streptavidin-Cy2 solution was applied and incubated for 30 min in the dark. Finally, slides were washed three times, air-dried and mounted with VectaShield mounting medium containing DAPI (Vector Laboratories, Burlingame, USA). Immunofluorescence was visualized by using a Zeiss fluorescence microscope and processed with the Zeiss AxioVison Rel. 4.8 (Zeiss, Jena, Germany).

### 4.6. Determination of Cell Viability Using Water Soluble Tetrazolium (WST-1 Assay)

SKOV-3 and OVCAR-3 were seeded in 96 well plates at a concentration of 3000 cells per well. After cells had attached culture media was changed to media containing either carrier solution, Gatipotuzumab (final concentration: 60 µg/mL), 4-OHT (final concentration: 5 µM) or the combination of Gatipotuzumab (final concentration: 60 µg/mL) and 4-OHT (final concentration: 5 µM). Cells were incubated for 48 h and 72 h, respectively. Following the stimulation period, 10 µL of WST solution (Roche, Mannheim, Germany) were added to each well. Plates were incubated for another 30 min before the plate was read on a microplate reader at 580 nm. Experiments were performed three times in different passages of cells achieving similar results.

### 4.7. Statistical Analysis Methods

Data were tested for statistical significance by employing the IBM statistic package SPSS (version 25) and by Microsoft Excel. SPSS was used to plot Kaplan–Meier graphs and box plots. Survival times were compared using log-rank statistics. Cell culture data were tested for statistical significance by using Student’s *t*-test. *p* values lower than 0.05 were considered significant.

## Figures and Tables

**Table 1:** Patients’ characteristics.

Cases with Data Available (*n*)	Characteristic	Category	n or median	%
138	Histology	serous	95	68.8%
		mucinous	12	8.7%
		endometrioid	20	14.5%
		clear cell	11	8.0%
133	FIGO	I + II	40	30.1%
		III + IV	93	69.9%
84	pN	neg.	37	44.0%
		pos.	47	56.0%
126	Grade	1	31	24.6%
		2 + 3	95	75.4%
137	age	≤60 y	73	53.3%
		>60 y	64	46.7%

Clinicopathological characteristics of the study sample are displayed. Numbers are displayed as raw count i.e., “n” (histology, FIGO, pN, grade) or median (age).

**Table 2:** TA-MUC1 as correlated to selected biomarkers stained on the same patient sample.

			GPCRs		Nuclear Steroid Hormone Receptors				Glycodelin			MUC1				
		GPER	LHR	FSHR	ERα	ERβ	PRA	PRB	Gd C15	Gd Q13	GdA	115D8	HMFG1	VU3C6	VU4H5	HER2
TA MUC1	c	0.177	0.095	0.087	0.230	0.131	0.100	0.009	0.219	0.214	0.066	0.366	0.458	0.479	0.353	0.012
	p	**0.038**	0.269	0.318	**0.007**	0.127	0.247	0.914	**0.010**	**0.012**	0.442	**<0.001**	**<0.001**	**<0.001**	**<0.001**	0.885
	n	138	136	134	138	136	135	137	137	137	137	133	136	136	134	136

TA-MUC1 was correlated to expression of G-protein-coupled receptors (GPCRs), nuclear steroid hormone receptors, glycodelin, MUC1, and HER2 by employing Spearman’s rho. TA-MUC1 was positively correlated to GPER, ERα, MUC1, and glycodelin. MUC1 was detected by different antibodies namely 115D8, HMFG1, VU3C6, and VU4H8. *p* values lower than 0.05 are shown in bold.

**Table 3:** Clinicopathological criteria of cases staining solely positive for TA-MUC1 vs. remaining cases.

		TA-MUC1/GPER			TA-MUC1/ER			TA-MUC1/GPER/ER		
		Remaining	TA-MUC1^pos.^ + GPER^neg.^	*p*	Remaining	TA-MUC1^pos^ + ERα^neg^	*p*	Remaining	TA-MUC1^pos^ + ERα^neg^ + GPER^neg^	*p*
Histology	other	37	6	ns	24	19	0.037	38	5	ns
	serous	78	17		35	60		82	13	
FIGO	I + II	39	1	0.004	20	20	ns	40	0	0.004
	III + IV	72	21		38	55		76	17	
pN	neg.	35	2	0.024	18	19	ns	36	1	0.021
	pos.	36	11		18	29		38	9	
Grade		31	0	0.004	19	12	0.007	31	0	0.011
		74	21		32	63		78	17	
Age ≤ 60 y		64	9	ns	30	43	ns	66	7	ns
Age > 60 y		50	14		29	35		53	11	

Positivity of TA-MUC1 staining, and at the same time loss of either GPER (TA-MUC1^pos^ + GPER^neg^) or loss of both ERs (TA-MUC1^pos^ + ERα^neg^ + GPER^neg^), characterized a subgroup of patients that were significantly more often staged as FIGO III/IV, graded as high grade and diagnosed as lymph node positive as compared to remaining cases. TA-MUC1^pos^ + ERα^neg^ was more common in tumors of high-grade or non-serous histology but was not otherwise related to clinicopathological parameters.

## Abbreviations

4-OHT	4-hydroxy-Tamoxifen
ER	estrogen receptor
FBS	fetal bovine serum
FFPE	formalin-fixed, paraffin-embedded
FIGO	Fédération Internationale de Gynécologie et d’Obstétrique
Gati	Gatipotuzumab
GPER	G-protein-coupled estrogen receptor
IHC	immunohistochemistry
MUC1	mucin-1
OC	ovarian cancer
OS	overall survival
PBS	phosphate buffered saline
REMARK	Reporting recommendations for tumor marker prognostic studies
TA-MUC1	tumor-associated mucin-1
